# The Doctrine of the Reflex-Function, in Its Relations to Physiology and Practical Medicine, Displayed and Critically Examined by Dr. J. W. Arnold

**Published:** 1843-07

**Authors:** 


### Art. X.

1. *Die Lehre von der Reflex-Function, für Physiologen und Aerzte, dargestellt und beurtheilt* von Johann Wilhelm Arnold, Doctor der Medicin, Chirurgie und Geburtshülfe, practischem Arzte in Heidelberg.—*Heidelberg*, 1842. 8vo, pp. 88.

*The Doctrine of the Reflex-Function, in its relations to Physiology and Practical Medicine, displayed and critically examined by* J. W. Arnold, Doctor of Medicine, &c.—*Heidelberg*, 1842.

2. *On the Diseases and Derangements of the Nervous System, in their primary forms, and in their modifications by Age, Sex, Constitution, Hereditary Predisposition, Excesses, General Disorder, and Organic Disease.* By Marshall Hall, m.d. f.r.s. l. and e. &c. &c.—*London*, 1841. 8vo, pp. 380. With Eight Copperplate Engravings.

The reflex theory of Dr. Marshall Hall has now been fully before the public for more than five years; and it appears to us that the time is come when its merits may be duly estimated. Ample opportunity has been afforded to those who had objections to adduce or criticisms to offer; and such have not been wanting. On the other hand, there has been time to subject many of the most important positions to the scrutiny of experience; and for this, too, there has been no lack of inclination, either amongst the supporters or the opposers of the theory.

We believe that we are justified in stating that, among the most eminent physiologists in this country, especially those who have made the nervous system the object of their especial study, there are few who do not receive Dr. Hall's views, at least in all their most important particulars. There may be a difference of opinion as to the degree in which these views had been glanced at by previous authors; but we believe that all are agreed in attributing to Dr. Hall the merit of originality, as to a large portion, and this the most important one of the doctrines advanced by him. The principal opponent in this country of that view of the action of the spinal cord which is peculiar to Dr. Hall is one for whose opinion we shall always entertain the highest respect; and did we not feel well convinced of the insufficiency of his objections, the weight of Professor Alison's authority would make us pause ere we ventured to take up a contrary position. But in Germany Dr. Hall's views are far from having gained as favorable a reception. By Professor Müller they are adopted to a certain extent; but there is, as it seems to us, so much obscurity and inconsistency in his disquisitions on this subject, that we are really at a loss to know his exact opinions. By several other German physiologists, however, objections have been raised to Dr. M. Hall's doctrines; and, so far as we can judge, the reflex-theory, as such, does not find much favour amongst them. We might be disposed, at first sight, to regard this circumstance as a serious argument against its correctness; since the authority of Valentin, Nasse, Volkmann, Arnold, and others of its opponents, on questions of neurology, stands deservedly high. But it is to be remembered that even Sir C. Bell's doctrines, respecting the distinct functions of the anterior and posterior roots of the spinal nerves, made but very slow progress in Germany, although they are now universally received there; and when we come to examine the objections adduced by Dr. Hall's opponents, we shall perhaps find that they are, after all, rather verbal than real.

We have, on former occasions, expressed our full conviction of the truth of the reflex-theory; and we shall now give evidence of the sincerity of this conviction, by taking up arms in its defence. The caustic and trenchant attack which has been made by Dr. Arnold, in the pamphlet before us, appears to us to call for special animadversion; since it embodies the objections brought by the various German physiologists we have alluded to, and brings them pointedly to bear on the question at issue; and also because it gives us the opportunity of exposing what we believe to be the radical error which pervades their whole train of reasoning on the subject. We shall first, however, give a sketch of the present state of the reflex-theory, and its applications to practice, as propounded by Dr. Hall in his latest work; and shall consider the most important difficulties attendant upon his views. Some of these may perhaps require modification in their detail; and such modifications we shall suggest, not in a captious, hypercritical spirit, but with the simple honest desire to render the system as stable and harmonious as possible.

The reflex-theory regards the true spinal cord as a centre of nervous action quite distinct from the encephalon; and as concerned in a set of actions of a peculiar and easily-defined character. That the cerebral hemispheres are the instruments of the intellectual operations is now universally admitted; in such operations, upon the reflex-theory, the true spinal cord has no participation whatever. The operations of the cerebrum are stimulated by *sensations* only; when they are taking place, we feel conscious of them, and we also feel a certain power of controlling and directing them by a strong effort of the will; and when they terminate in producing a muscular contraction, it is by an exercise of *volition.* Thus, food is placed before us; we become conscious of its proximity by sensation; our judgment is exercised in determining whether the food is desirable, wholesome, and palatable; and, if an affirmative decision is formed, a voluntary contraction is produced in certain muscles, by which it is carried into execution. Hence, the cerebral hemispheres must be directly connected with almost all parts of the system; since there are few parts not capable of receiving sensory impressions, and nearly the whole muscular apparatus is under its control. This connexion is established by means of the nervous trunks, some of which convey the sensory impressions to the sensorium, whilst others carry the mandates of the will to the muscles, and excite contractions in them. Hence we may very properly term the nervous trunks proceeding from and towards the hemispheres, *sensori-volitional* fibres.

Now these fibres exist, not only in the nerves of special sense, and other cephalic nerves, but in every nerve that is given off from the spinal cord; for this organ consists, in part, of a mere collection of fibres, which are continuous with those of the nervous trunks, and which establish the communication between these and the brain. The difference between the *true* spinal cord and the compound structure generally known under that name, is apparent at its lower part, *cauda equina.* The true spinal cord does not usually extend in man below the first lumbar vertebra; and the canal is occupied, below that point, by a bundle of nerves, which is but a continuation of a large part of the white or fibrous structure in the upper part of the medulla. The *true* spinal cord is entirely distinct from this, as distinct as are the ganglia of the nervous column of the articulata, from the fibrous tract which passes over them. It is, in all essential particulars, a true ganglionic centre, having functions peculiar to itself; and this is clearly established by the fact, that motions dependent upon it are performed after the brain has been removed, or the connexion with it interrupted. That the nervous fibrils which are connected with the spinal cord are *anatomically* distinct from those which are connected with the brain, though bound up in the same trunk with them, and distributed to the same organs and tissues, is a doctrine which seems to follow naturally from Dr. Hall's proofs of their *physiological* distinctness; but it has been repeatedly disavowed by Dr. Hall as a part of *his* system, and it cannot be fairly attacked, therefore, by Dr. Hall's opponents, as one of his positions.

The doctrine of the structural distinctness of the spinal and cerebral systems of nerves was first advocated by Mr. Grainger, in his Treatise on the Spinal Cord. It was adopted by Dr. Carpenter, as the result of his comparative researches into the structure and physiology of the nervous system in the articulated and molluscous classes. And it now derives strong confirmation from the recent inquiries of Mr. Newport on the myriapoda.* He finds that the fibres which correspond to those of the true spinal cord in vertebrata are perfectly distinct from those connected with the cephalic ganglia. They form part of the cord in the intervals between the abdominal ganglia; and may be traced from the periphery into the several ganglionic centres, from which they pass backwards along the cord until they arrive at the next ganglion, from which they pass again to the surface of the body. The function of these fibres cannot be other than reflex; and thus is anatomically proved that which Dr. Hall contended for physiologically, and that which Dr. Carpenter had inferred from other forms of structure. Mr. Newport's experiments (of which, through his kindness, we shall be able to give some account,) seem to prove most decidedly that the supra-œsophageal ganglia in articulata are the seat of sensation and volition; and that, when these are removed or greatly injured, all the movements of the body are reflex.

The peculiarity of the action of the spinal cord, according to Dr. Hall, consists in this; that whilst *sensation* is required to prompt the operations of the intellect, to excite emotions, or to stimulate instincts to action, *no sensation* is necessary to produce those actions which are performed through this organ; but that the *impression* made upon the peripheral extremity of a nerve, and brought by it to the spinal cord, may excite a reflex muscular action, which shall succeed it without any *necessary* consciousness on the part of the individual, and therefore, without any mental operation whatever. In Mr. Newport's paper, although there is no direct *proof* that the ganglia are *not* sensitive, there is strong inferrential evidence that they are not so; since, although reflex acts of locomotion may be repeatedly exerted in the body after removal of the head or destruction of the central ganglia, these are always performed in precisely the same manner, and without any evidence of volition. In the

* "On the structure, relations, and development of the nervous and circulatory systems, and on the existence of a complete circulation of the blood in vessels, in the myriapoda, and macrourous arachnida." Communicated to the Royal Society, April 6, 1843.

ordinary condition of the system, however, consciousness *does* accompany these actions; but for this simple reason, that the impression which excited the spinal cord to action through its nerves, is also conveyed to the brain, and excites *it* to action through *its* nerves. But, if the excitement of a cerebral action be prevented, either by division of the spinal cord in its upper part so as to cut off communication with the brain, or by a state of inactivity in that organ, as in profound sleep and coma, no sensation is produced, yet the reflex actions go on as before. Some reflex actions take place, however, in the natural condition of the body, without sensation; this is the case, for instance, with regard to the propulsion of food along the œsophagus, which has been shown by Dr. J. Reid to be a reflex action; yet, after the food has passed the pharynx, there is no sensation, except such as is produced by its pressure on the surrounding parts, or by an irritation which its heat or acridity may produce in the mucous membrane. The convulsive movements in disease, again, are frequently excited by causes which do not produce sensations,—a circumstance on which we do not think enough stress has been laid by the upholders of the reflex theory. But the most decisive evidence of the non-participation of sensation, as a necessary link in the chain of reflex actions, is derived from the cases (of which many have now been subjected to careful examination) of disease or injury of the spinal cord in the human subject, cutting off the connexion of the lower extremities with the brain, but not impairing the reflex power of the lumbar portion of the cord; in the lower extremities of such persons, distinct and vigorous actions may be produced by slight irritations, which are not in the least degree felt by the patient. Indeed, it would seem that, when the muscles are thus withdrawn from cerebral control, they are much more readily excited to reflex action, than when they are under the direction of the will excited by sensation.

These facts are admitted by Dr. Alison; but he does not go along with Dr. M. Hall in the inferences he draws from them. "It is certainly possible," says Dr. Alison, (Outlines of Physiology, p. 211,) "that impressions made on sensitive nerves in the living state, but when the power of sensation is suspended, may so imitate those changes in them, which in the natural state are attended by sensations, as to produce some of the muscular contractions by which these sensations are generally succeeded and indicated; and all that can be certainly inferred from the facts in question is, that it is in the spinal cord that this *crossing* of the action excited in the nervous matters from the sensitive to the motor filaments takes place. But there is no distinct evidence that *regular combinations and successions* of muscular movements can be effected in distant muscles by impressions on sensitive nerves, without sensation intervening as part of the chain of events." The movements of respiration and deglutition are regarded by Dr. Alison as coming under this last category. Now on this we take leave to remark that, in our opinion, the burden of proof lies with those who maintain that sensation *does* participate in these movements, and that the spinal cord does possess, in any part of it, when isolated from the brain, the property of *sensibility*, by which we mean the power of rendering the individual *conscious* of the impressions conveyed to it. We submit that the very same kind of argument which Dr. Alison adopts (and in our opinion most correctly) to prove that secretion and nutrition are not dependent upon nervous influence, will, if fairly carried out, necessarily lead to the conclusion that the reflex actions of the spinal cord are not dependent upon sensation. For it can be *proved* of many of these actions, that sensation has no possible participation in their production; therefore the existence of *reflex action without sensation*, must be admitted as a physiological fact. Moreover, it must be admitted that the lower portion of the spinal cord is not a distinct centre of sensation, when separated from the upper. It is for those, therefore, who maintain that the medulla oblongata may become a distinct centre of sensation, when separated from the brain, and that the actions performed through it involve sensation as a necessary condition, to prove their position; and we feel justified in stating that nothing like *valid proof* has ever been given. A great deal too much reliance has been placed upon the *adaptive* character of many reflex actions, as indicative of sensation; but what can be better adapted to the ends they are destined to accomplish than many movements of plants and animals, in which it is not supposed by any that sensation necessarily participates. To go no further than our own bodies; the contractions and dilatations of the heart, the peristaltic motions of the alimentary canal, and the similar movements of the fallopian tubes and of the ducts of the principal glands, exhibit a most obvious adaptive character, though performed without our consciousness; and there are well-known instances on record, in which the act of parturition and the ejaculatio seminis have taken place, when the sensibility of the genital organs had been entirely destroyed by section or disease of the spinal cord. This observation of Dr. Alison seems now likely to be put aside by the experiments and observations just referred to; in which Mr. Newport found that *regular combinations and successions of muscular movements in distant muscles can be excited in the myriapode, after decapitation*, or after removal of the cerebral ganglia without decapitation, by irritating the cut extremity of the cord, by pressure applied to the upper surface of a segment of the body, or by irritation of individual ganglia. By any of these means, acts of locomotion may be repeatedly excited after decapitation; and the body is moved forwards by regular and successive actions of the legs as in the natural state, but these movements are always directly *forwards*, *never* backwards, and rarely to either side, and then only when the direct line of progression is impeded on one or other side, thus giving mechanically a different direction to the body. These motions, in a newly decapitated individual, are at first almost as vigorous as in the natural state, but gradually become slower and slower until they are so feeble as not to carry the body forwards, and are then continued for some time longer until they entirely cease. On re-application of the stimulus after a few minutes of complete rest, these movements may be re-excited and the body carried forwards as before. But in these movements there is not the slightest indication of consciousness, no direction of object, no avoidance of danger. If the body be opposed in its progress by an obstacle not more than one half its own height, it mounts over it and moves directly onwards, as in a natural state; but if the obstacle is equal to its own height its progress is arrested, and the cut extremity of the body opposed to it remains in contact, with the legs still moving as in the act of locomotion. Who can doubt that these motions are entirely reflex, and devoid of what we regard as sensation?

Notwithstanding the great stress laid by some writers, on the medulla oblongata as a peculiar centre of nervous agency, we quite agree with Dr. Hall in regarding its sole peculiarity to consist in the nature of the actions to which it ministers; these actions not being, like those of the spinal cord, movements of general locomotion, but being intimately connected with the maintenance of the organic functions. The study of comparative anatomy makes this very evident. For in the mollusca and articulata we find the respiratory and pharyngeal ganglia quite distinct from the pedal or locomotive; and the former are placed within the cranium of vertebrata, obviously because they are there more secure from injury.

It is quite true that there *are* actions which, though not voluntary, are still dependent upon sensation as one link in the chain. To these, perhaps, the term *instinctive* might be properly restricted. Some of them seem to require the sensation merely; such are the corresponding or *consensual* movements of the two eyes. In others, something like an emotion takes place; as in the production of laughter by tickling. And in others, the emotion is still more decidedly a necessary link; as when laughter results from the excitement of ludicrous ideas. And we freely admit that it is not always practicable to determine whether a particular action should be referred to this class or to the preceding. Thus, although we do not hesitate in regarding the ordinary acts of inspiration and expiration as of a purely reflex character, we think it still doubtful whether those peculiar combinations of muscular movement which constitute coughing and sneezing are not excitable by sensation only. But if the *principle* of reflex action without sensation be recognized, a difference of opinion on such details may for the present be overlooked.

We shall now briefly follow Dr. Hall through his exposition of the anatomy, physiology, and pathology of the *cerebral* and *spinal* systems, as set forth in the volume before us.

The *cerebral* system consists of the cerebrum, with the two sets of nervous fibres connected with it. One of these consists of fibres which originate in the sensory surfaces of the body, and terminate in the cerebrum, whilst the other consists of those which originate in the cerebrum, and convey the influence of volition to the muscles. All parts which are sensible to impressions, and which can be affected by the will, are thus connected with it; although the same parts may, by another set of fibres, be connected with the spinal cord. The posterior roots of the spinal nerves, and those of the fifth and eighth pairs of cerebral nerves, must be regarded as compound in their structure; containing both sensory fibres which proceed to the brain, and excitor fibres which terminate in the spinal cord. And in like manner the anterior roots of the spinal nerves, with the third and fourth small root of the fifth and sixth portio dura of the seventh, and motor portion of the eighth and ninth nerves, must be regarded as having a double origin in the brain and spinal cord, being subservient to the voluntary motions directed by the former, and to the reflex actions executed by the latter. "Every compound sentient and excitor, and voluntary and motor nerve," says Dr. Hall, "must be presumed, physiologically speaking, to have *two connexions, one cerebral, the other spinal*, with sentient and excitor *origins* in cutaneous and mucous surfaces, and appropriate *destinations* in the muscular system." (§ 104.) In this and several other passages, Dr. Hall speaks of the *physiological* distinctness of the cerebral and spinal systems; and guardedly avoids any intimation of his opinion of their *structural* distinctness. Yet we must own that this appears to us a distinction without a difference. For the question at last reduces itself to this :—are the reflex actions, and the sensori-volitional actions performed by the same or by different fibres? If by the same, can we say (consistently with our knowledge of the elementary structure of the nervous system) that any *single* fibre can have *two* origins or terminations in the central and in the peripheral organs respectively? Can *a single* afferent fibre, for instance, have *two* origins, a sensory and an excitor, in a mucous surface, and *two* terminations, one of the brain, the other in the spinal cord? or can a single motor fibre have *two* origins, one in the brain, the other in the spinal cord? If it be admitted that these actions are performed by different fibres, then why not say so? It is true that their structural distinctness cannot be clearly made out in vertebrata; but in many of the invertebrated tribes there is not the same difficulty. And it is interesting to remark that whilst, in the vertebrata the fibres of the spinal nerves are so arranged that the afferent, both sensory and excitor, are bound up in one set of roots, and the motor, both volitional and reflex, in the other,—the arrangement of the fibres in the articulata and mollusca is such, that one set of roots of each nerve contains both the afferent and efferent fibres of the sensori-volitional system, and the other the excitor and motor fibres of the reflex system; as if nature had intended to give us the opportunity of discovering the isolation of all these systems, by the extension of our survey throughout her domain. If we had been confined to the study of the articulata, we should have discovered the structural distinctness of the sensori-volitional and the reflex systems; but we should have had no clue to the distinction between the afferent fibres, which convey impressions from the periphery to the centre, and the efferent or motor fibres, which convey the motor impulses from the central organs of the muscles. On the other hand, by the exclusive study of the vertebrata, we *have* discovered the latter, but not the former.

"The physiology of the cerebral system," says Dr. Hall, "comprises sensation in all its forms, perception, judgment, volition, and voluntary motion." (§ 106.) "The distinctive character of a voluntary motion is its spontaneous occurrence. Without sensation, without emotion, the individual *wills*, and a voluntary movement succeeds." (§ 112.) We do not consider this to be the really distinctive character of voluntary motions. The exercise of the will is consecutive upon the other processes enumerated by Dr. Hall, and these are at first stimulated by sensation. If we could conceive a being to come into the world endowed with a perfect brain and every mental faculty complete, but all the inlets of sensation closed, there would be no mental *operation*: for it is *sensation* which is the necessary stimulus to any change in the mental condition. The being would remain in a torpid state, like a man in profound sleep, possessing faculties, yet wanting the power of using them; just as the seed buried deep in the soil possesses vitality but does not germinate, because withdrawn from the influence of the requisite stimuli. But when the mind has been once aroused by sensation, it is rendered permanently active; for the sensation does not produce a merely transient change, but is like the spring, by touching which we set a complex machine in continued operation. The sensation is retained and may be reproduced by the act of conception at any remote time, or it may spontaneously recur to the mind; and thus all the actions to which it gives rise may be in appearance spontaneous, whilst they are in reality as dependent upon sensations, felt or remembered, as are those of a reflex nature. The real distinction appears to us to be this. *Voluntary* motions are executed only as means to a certain end which the mind has in view; in other words, we throw a certain set of muscles into action, in order to accomplish a certain determinate object. In the reflex and instinctive actions, on the other hand, there is no perception of the object, or choice of means. But there is this remarkable peculiarity about voluntary motions, to which we do not think that enough attention has been given. We *will* to perform a certain movement, but we do not determine by the will the muscles which are to execute the movement. We must have the end or purpose of the operation in our mind, to constitute it a voluntary movement; but the immediate influence on the muscles is something different from the will, and approaches more to the character of an intuitive direction. We shall first take for illustration the case of the muscular operation concerned in the production of vocal sounds or musical tones. A little consideration makes us aware that we cannot *voluntarily* utter a given sound or tone, without in the first instance conceiving it (however transiently) in our minds; and the particular operation upon the muscles necessary to produce it is certainly not voluntary, for our *judgment* could not select the precise strain on each which is requisite to produce the combined effect. But an *emotional* cry is performed from the mere instinctive tendency, without any such previous conception. We believe that all persons whose organs of voice are naturally formed can at once execute any tone of which they form a definite conception; for singing out of tune is well known to be due to an imperfect musical ear, which renders that conception weak and indefinite. But whether the intuition is original or acquired in infancy we shall not at present discuss; probably there is some variation in this respect. Now this is rather an extreme case; and we have purposely selected it, because the remoteness of the relation between the muscular action and the effect to be produced, renders the distinction between them sufficiently obvious. But this distinction is not less valid in other cases. All the muscular movements which we call voluntary are under the *immediate* guidance of *sensation*, either received from the muscle itself or from some other source. The case of the woman whose arm was affected with anesthesia, and who could not continue to hold up her child unless she looked at it, is a most satisfactory proof of this; and the case is not a solitary one. The muscular sense being destroyed, even the strong voluntary effort could not keep up contraction in a certain set of muscles without the guidance of the visual sensation. Hence we see that there is something intermediate between the will and the muscles, of which the ordinary theory of voluntary motion does not take cognizance; and this might be inferred also from the fact, that irritation of the hemispheres does not excite muscular movements, though these are immediately shown when the irritating cause operates on the tubercula quadrigemina or on the medulla oblongata.

We return from this digression to the proper functions of the cerebrum, in which Dr. Hall includes the *emotional* actions. These he regards as distinguished from the voluntary, in being *immediately* excited by sensations. "Thus a disgusting object placed before the eyes will induce vomiting and fainting." But he considers that, though their seat is in the cerebrum, and though they depend upon sensations, they operate through the true spinal system of nerves. We must own ourselves unable to comprehend how the true spinal nerves, which terminate and originate in the true spinal cord, can be the ministers of changes which take place in the brain; and it seems to us that the same line of argument which establishes the distinctness (whether we prefer to call it structural or physiological) of the true spinal or reflex, and of the cerebral or volitional systems, must also establish the existence of the emotional as distinct from either. Dr. Carpenter has brought forward a theory on this subject which we believe to be new, and to be supported by much anatomical evidence. (Human Physiology, §§258-265). He identifies the *emotional* actions with that restricted class of the *instinctive* which involve sensation (the lower instinctive actions being merely reflex), and localizes them in the ganglia of special sense, which constitute nearly the whole cephalic mass of the invertebrata, and which in fishes and reptiles (whose intelligence is very low) bear a very large proportion to the cerebral hemispheres. In mammalia, the greatly-increased size of the hemispheres throws these ganglia, as it were, into the shade, but still they exist; and we find that from the corpora quadrigemina and the neighbouring ganglionic substance, into which we may trace the chief terminations of the optic, olfactory, and auditory nerves, there issues a set of fibres which pass down through the corpora olivaria into the anterior columns of the spinal cord, and thence into the motor roots of the spinal nerves. The small size of these ganglia in man, and the small proportion which their motor fibres bear to those which originate in the cerebral hemispheres, is exactly what we should anticipate from the small proportion which his purely emotional and instinctive actions bear to those which are dictated and guided by his intelligence.

The cerebral system is inactive during sleep; and to it alone is due the sense of fatigue. Its diseases produce the effects which we should anticipate from its normal functions.

"Every derangement of the senses, every form of delirium or of coma, or of perverted imagination or judgment, every act of violence, must be referred to the condition, primary or secondary, of the cerebrum or cerebellum." (§ 120.) When the cerebrum is irritated, delirium ensues. When compressed, coma is induced. When lacerated, we have paralysis of *voluntary* motion. If other phenomena are seen in diseases of the encephalon, they arise from the extension of the influence of these to the true spinal and ganglionic systems, through *irritation* or *pressure*, *counter-irritation*, or *counter-pressure*; points of extreme importance, to be noticed very particularly hereafter." (§ 135.)

The connexion of disordered action of the cerebral system with disordered circulation is thus concisely stated by Dr. Hall:

"Not only undue arterial action and venous congestion induce morbid states of the cerebral functions, but the state of exhaustion from loss of blood, the anemious condition in chlorosis, &c. induce *similar* effects, and present to the physician anxious cases which frequently try his skill in diagnosis. Too great action of the minute arteries, congestion in the veins, an anemious state of the vascular system of the encephalon, alike induce morbidly exalted and impaired conditions of the mental and cerebral functions. Spectra, delirium, insomnia, amaurosis, stupor, coma, violent voluntary actions, or paralysis of the voluntary motions, these are the symptoms which arise out of these morbid conditions of the cerebral system and functions, and these only.

Spasmodic actions depend upon the fact of another system being implicated." (§§ 140-142.)

Dr. Hall's account of the therapeutics of the cerebral system is very brief. He confines himself to the indication of the fact that there are substances, such as green tea, alcohol, opium, &c. which have a specific effect upon the cerebrum; and to the quotation of M. Flourens' researches on this subject.

We next proceed to the true spinal or excito-motory system, of whose functions we shall give Dr. Hall's general account in his own words:

"1. Its anatomy involves a system of incident and reflex nerves connected with the true spinal marrow as their centre, unknown in this special connexion before. 2. Its physiology consists in functions, all of which are performed through this peculiar anatomy. These functions comprise all the acts of ingestion, of retention, of expulsion, and of exclusion in the animal economy; they are those therefore on which depend, 1st, the preservation of the individual, and, 2d, the continuance of the species. All these functions are reflex-spinal functions, of which the idea did not formerly exist. 3. Its principle of action is the *vis nervosa* of Haller, a motor power of which there was previously no application to physiology whatever. 4. Its pathology comprises the whole class of spasmodic diseases and its subdivision into those of, 1st, incident; 2d, centre; 3d, reflex, origin. 5. Its therapeutics are similar to its pathology; the various physical agents, especially severe cold, sometimes acting on the incident nerves, sometimes on the spinal marrow itself, sometimes on the reflex nerves." (p. 39.)

We have on former occasions expressed our want of accordance with Dr. Hall, in regard to the importance which he attaches to the *third* of the preceding principles, and also with respect to the absence of any preceding ideas of reflex action. We shall not therefore now revive these discussions; but simply express our full and hearty assent to the claim which he advances, of having entirely elicited this system, as a system, by his own laborious and persevering researches. The following is Dr. Hall's own tabular view of the *anatomy* of this system:

I. *The Incident or Excitor Branches.*

i. The Trifacial, arising from

 1. The Eyelashes.

 2. The Alæ Nasi.

 3. The Nostril.

 4. The Fauces.

 5. The Face.

ii. The Pneumogastric, from

 1. The Pharynx.

 2. The Larynx.

 3. The Bronchia.

 4. The Cardia, Kidney, and Liver.

iii. The Glosso-pharyngeal?

iv. The Posterior Spinal, arising from

 1. The General Surface.

 2. The Glans Penis vel Clitoridis.

 3. The Anus.

 4. The Cervix Vesicæ.

 5. The Cervix Uteri.

II. *The True Medulla Oblongata and Medulla Spinalis.*

*The Centre of the System.*

III. *The Reflex or Motor Branches.*

i. The Trochlearis } Oculi.

ii. The Abducens } Oculi.

iii. The Minor Portion of the Fifth iv. The Facial, distributed to

 1. The Orbicularis.

 2. The Levator Alæ Nasi.

v. The Pneumogastric, or its Accessory.

 1. The Pharyngeal.

 2. The Œsophageal & Cardiac.

 3. The Laryngeal.

 4. The Bronchial, &c.

vi. The Myo-Glossal.

vii. The Spinal Accessory.

viii. The Spinal, distributed to

 1. The Diaphragm.

 2. The Intercostal and Abdominal Muscles.

ix. The Sacral, distributed to

 1. The Sphincters.

 2. The Expulsors, the Ejaculators, the Fallopian Tubes, the Uterus, &c.

The succeeding table conveys at a glance a complete view of the physiology of the true spinal system, according to Dr. Hall.

I. *The Excited Action.*

i. Of the Eyes, the Eyelids, (and of the Iris?)

ii. Of the Orifices { 1. The Larynx. 2. The Pharynx.

iii. Of the Ingestion.
- 1. Of the Food.
  - *a.* In Suction;
  - *b.* In Deglutition.
- 2. Of the Air, or Respiration.
- 3. Of the Semen or Conception.

iv. Of Exclusion.

v. Of the Expulsors or of Egestion.
- 1. Of the Fæces.
- 2. Of the Urine.
- 3. Of the Perspiration.
- 4. Of the Semen.
- 5. Of the Fœtus or Parturition.

vi. Of the Sphincters.
- 1. The Cardia.
- 2. The Valvula Coli?
- 3. The Sphincter Ani.
- 4. The Sphincter Vesicæ.

II. *The Direct Action or Influence.*

i. In the Tone } of the Muscular System.

ii. In the Irritability }

We think the last subdivision the most objectionable of the whole; for it may be doubted whether the spinal system ever exercises the direct influence attributed to it by Dr. Hall. We have expressed ourselves on several former occasions, as quite opposed to the belief that the *irritability* of the muscular system has any relation of dependence upon any part of the nervous system; and the facts which Dr. Hall regarded as indicating such a dependence have been shown by Dr. John Reid to be susceptible of, and indeed to require, a very different explanation.* And in regard to the *tone* of the muscular system, we think it may be questioned whether this is not dependent upon a reflex, rather than upon a direct action of the spinal cord. The following experiments prove the influence; its nature remains uncertain:

"Two rabbits were taken: from one the head was removed; from the other also the head was removed, and the spinal marrow was cautiously destroyed by a sharp instrument; the limbs of the former retained a certain degree of firmness and elasticity; those of the second were perfectly lax. The difference was most obvious....The limbs and tail of a decapitated turtle possessed a certain degree of firmness or tone, recoiled on being drawn from their position, and moved with energy on the application of a stimulus. On withdrawing the spinal marrow gently out of its canal, all these phenomena ceased. The limbs were no longer obedient to stimuli, and became perfectly flaccid, having lost all their resilience. The sphincter lost its circular form and its contracted state, becoming lax, flaccid, and shapeless. The tail was flaccid, and unmoved on the application of stimuli."

It would be an interesting experiment, to divide the *posterior* roots of

* See Edinburgh Monthly Journal, May, 1841; and Brit. and For. Med. Rev., vol. XII. p. 277.

all the spinal nerves, instead of removing the cord. If, as we rather anticipate would be the case, the tone of the muscles were then lost, it would be evident that this state of moderate contraction is the result of a reflex action of the cord, excited by some impression conveyed from the muscle; and not a direct influence originating in the cord itself. We have thrown out this hint, merely for the purpose of simplifying the system; because it appears to us more likely to operate on *one* general principle, than in two distinct methods.

For a most luminous and interesting account of the various subordinate divisions of the reflex function, and of the nerves concerned in each, we must refer our readers to Dr. Hall's work; as it would not be consistent with our plan to go through these in detail. There are few physiological principles which are more susceptible of varied and important applications than those which he has here developed; and we earnestly recommend all our readers, who have not already done so, to make themselves well acquainted with them. There is no department of pathology which is capable of being so much improved by a good physiology as is that of the nervous system; and Dr. Hall's researches have not only pointed the way, but have carried us far towards our ultimate goal.

We must commence this part of the subject, by observing that the tables already quoted do *not* express the *whole* anatomy and physiology of the spinal system, but the most important aspects under which it is to be viewed, as ministering to the *organic* functions in man. Its operations appear to be in him more limited to the supply of the conditions requisite for the performance of these, than they are in the lower animals, with whose locomotive actions it often seems intimately connected. Thus it is probable that the regular movements of the wings of birds and insects are rather of a reflex than of a voluntary character; indeed they must be, if we admit that the cerebrum only is the centre of volition, since they will continue after its removal. The locomotive actions of man are under the complete control of his will; and those muscles of his general system which are not connected with the organic functions are only incidentally affected by reflex agency. But they must be all supplied by the spinal system of nerves; since we see that, in disordered conditions of that system, they are as strongly affected as any. Yet the groups of muscles connected with respiration, deglutition, and egestion, are the most liable to suffer from its disorders, as will be seen from the following table:

PATHOLOGY OF THE TRUE SPINAL SYSTEM.

I. *Diseases of the Incident Nerves.*

| | | |
|---|---|---|
| I. 1. Dental<br>2. Gastric<br>3. Intestinal | irritation in infants. | 1. The crowing inspiration.<br>2. Strabismus, spasm of the fingers and toes, strangury, tenesmus, &c.<br>3. Convulsion.<br>4. Paralysis? |
| II. 1. Gastric<br>2. Intestinal<br>3. Uterine | irritation in adults. | 1. Hysteria.<br>2. Asthma.<br>3. Vomiting, hiccup, &c<br>4 Epilepsy.<br>5. Puerperal convulsion. |

III. Traumatic tetanus, hydrophobia, &c.

II. *Diseases of the Spinal Marrow itself.*

i. Inflammation and other diseases.
ii. Diseases of the vertebræ and membranes.
iii. Counter-pressure, &c. in Diseases within the cranium.
iv. Centric epilepsy, tetanus, &c.
v. Convulsions from loss of blood, &c.

III. *Diseases of the Reflex or Motor Nerves.*

i. Spasm.
  1. Spasmodic tic.
  2. Torticollis.
  3. Contracted limbs, &c.
ii. Paralysis.

In epilepsy, tetanus, hydrophobia, hysteria, and the crowing inspiration of children, the symptoms are all of the same character; and the chief difference consists in the parts affected. Thus in *epilepsy*, besides the general convulsion, the expulsors are chiefly excited; and there is frequently at the same time spasmodic closure of the glottis, so that, in spite of violent expiratory efforts, the air is retained within the chest, the face becomes livid, and death may take place during the fit. *Tetanus* is characterized by the continued spasm of the muscles of the back, neck, and jaw. The muscles of deglutition are specially affected; and death usually results from the *fixation* of the respiratory muscles, so that asphyxia takes place. In *hydrophobia,* there is laryngeal as well as pharyngeal spasm; and death results from the closure of the glottis. In this disease, the emotional system of nerves seems to be more affected than in the preceding. The patient expresses a peculiar horror at the sight or sound of fluids; and the spasmodic action is brought on by *sensations,* more than by internal impressions. In that proteiform and perplexing disease, *hysteria,* almost all the varieties of spasmodic action are exhibited, even by the same patient at different times. Dr. Hall considers it a well-marked character of difference between epilepsy and hysteria, that, in the latter, however much the larynx may be affected, it is never closed; in epilepsy it is closed: in the former we have heaving, sighing, inspiration; in the latter violent ineffectual efforts at expiration, (§ 1679.) We have for some time had under our observation, however, an aggravated case of hysteria, which has exhibited this epileptic symptom in a very alarming degree; the face having often become quite livid, before the spasm of the glottis yielded. Dr. Hall might say that this symptom was indicative of epilepsy; but the case has nothing else in common with that disease, and has put on by turns all those variations which are so characteristic of aggravated hysteric disorder. It is one, too, in which the *emotional* character of this disease has peculiarly manifested itself. Dr. Hall very justly says that "hysteria seems to single out and affect every organ, every function which belongs to the true spinal system." Like the emotions, it also affects the action of the heart, the secretions, especially that of the kidney, &c. The *crowing inspiration* or croup-like convulsion of children bears a strong resemblance to epilepsy in the mode in which the muscles are affected. Its chief symptom is, as its name imports, a partial impediment to the inspiratory action; but as soon as this impediment increases, so as nearly to close the larynx, strabismus and general convulsions come on, with violent expiratory movements, and not unfrequently an affection of the sphincters.

This reference of all convulsive diseases to the spinal (including the emotional) system of nerves alone, we believe to be one of the most important advances which the pathology of the nervous system has yet made; and the idea of it is due entirely to Dr. Hall. Not unfrequently the source of these diseases may lie within the cranium, or may be even in the brain itself; but it cannot act upon the muscular system, so as to produce convulsions, unless it affect, in some of the methods already indicated by Dr. Hall, either the tubercula quadrigemina, (the centre of Dr. Carpenter's emotional system,) or the medulla oblongata, or some other part of the spinal nerve. The experiments of Magendie and others, who have shown that no irritation of the cerebrum proper can directly produce muscular motions, form the basis of this view; which is borne out by a number of other physiological and pathological phenomena. A frequent source of convulsive action from cerebral disorder is undoubtedly pressure or irritation of the nerves of the dura mater, which is supplied by the fifth pair, and which is thus connected with the spinal system; for Dr. Hall has found that on pinching the dura mater of an animal just killed, violent convulsive movements were excited. It remains for intelligent practitioners to reobserve, on Dr. Hall's improved plan, the phenomena of all forms of nervous disease; and we shall be happy if, by this notice of Dr. Hall's views, we can excite more general attention to the subject. For the application of his principles to each particular form of disease, our readers must consult the work itself; which ought to have a place in every medical library. The diagrams with which it is illustrated are very ingenious; and will, to many, afford considerable aid in the comprehension of the text.

We have now the less pleasing task of laying before our readers an account of Dr. Arnold's brief treatise. The object of the writer (who is not *the* Arnold of neurological fame, but a brother possessing a much less extensive renown,) is evidently to upset the whole doctrine of the reflex function, as propounded by Dr. Marshall Hall; for he labours throughout to prove that what is new in that system is not true, and that what is true is not new. With this view, he brings together the facts and opinions bearing on the question, which had been promulgated by various older physiologists; and intimates that Dr. M. Hall and Professor Müller might have spared themselves much trouble, if they had been better acquainted with the labours of their predecessors. As we have fully gone over this ground on a former occasion, (vol. V, pp. 523-40,) and as we do not meet in Dr. Arnold's resumè, with any facts of importance which had been overlooked by us, (indeed we think he might have profited much by consulting *our* retrospect,) we shall dwell but lightly on this part of his inquiry; directing our attention rather to the arguments he adduces against the system as it now stands.

We cannot but remark, throughout this disquisition, a very loose mode of reasoning, both when the author is attacking the position of his opponents, and when he is endeavouring to establish his own. And though there is considerable ingenuity in many of his objections, yet they are often brought to bear, rather against his own inferences from the reflex doctrines, inferences which its chief supporters would probably not acknowledge, than against the fundamental dogmas of the system itself, as laid down by its author. Of this we shall presently meet with more than one example. There appears to us, also, to be a great deficiency of precision and clearness in the use of abstract terms; and this fault, we may hint, is common to most German writers on physiology. They get on very well so long as their language applies to tangible things; but so soon as they quit the palpable they become obscure. We suspect that this fault has a good deal to do with the dreamy mysticism in which some of the most applauded among the German school of philosophy delight to indulge. We have often thought it a curious anomaly that men on the one hand so laborious in the accumulation of physical facts and in the investigation of their causes, and on the other so prone to indulge in metaphysical reasoning of the most abstruse kind, should be so deficient in the power of conceiving definitely, and applying correctly, the simple principles by which groups of phenomena are at once expressed, by which the investigation is conducted to new truths, and which alone can be relied on as guides in the application of physiology to practice. Yet so it unquestionably is; for though it is the fashion to cry up German physiology as immeasurably in advance of British,* we think it would not be difficult to show that in a truly *scientific* view, we are, and long have been, greatly in advance of our continental neighbours; our physiological facts being more completely reduced under the dominion of principles; and these principles being at once more definite in their character, and more precise in their character, than those which have held their sway in Germany. To draw an illustration from the present subject, which affords many such. From the time of Whytt, the doctrine taught in the University of Edinburgh respecting the sympathetic motions has been, that they are dependent upon the cerebro-spinal system; and that they result from the conveyance of a stimulus from the peripheral extremities of certain nerves to the central organs, where it excites an impulse which is transmitted downwards to the muscles. Dr. M. Hall has recently shown that no other part of the central organs is required for this purpose than the spinal cord; and he has also shown that sensation is not a necessary link in the chain of actions, as maintained by some. Now, with the exception of a few stray writers, like Prochaska, whose more correct opinions never seem to have excited any general attention, the almost universal opinion of German physiologists down to the time of Müller appears to have been, that the sympathetic movements are dependent on the sympathetic system of nerves, a doctrine which, as we need hardly tell our readers, is now quite exploded. But instead of receiving the simple and definite conceptions now presented to them by Dr. M. Hall, many of the German physiologists, and our author among the rest, flounder about in a slough of unmeaning, because indefinite, generalities; which after all seem to us to come much nearer to the doctrines they reject than their authors imagine.

The difficulty under which the physiologists of the German school labour in this matter is chiefly one of words; and as a great deal hinges on the meaning attached by them to the terms *sensation* (empfindung) and *perception*, we shall briefly examine it. Among British metaphysicians and physiologists the meaning of the former term has long been definitely fixed. They are all agreed, we believe, in regarding consciousness as a necessary element in sensation. Dr. Brown (in arguing against the doctrine of Dr. Reid, that consciousness is a distinct attribute of the mind)

* See recent articles in the Lancet.

thus expresses himself. "If, immediately after the first sensation, we imagine the sentient principle to be extinguished, what are we to call that feeling which filled and constituted the brief moment of life ? It was a simple sensation and nothing more; and if only we say that the sensation has existed, whether we say or do not say that the mind is conscious of the sensation, we shall convey precisely the same meaning; the consciousness of the sensation being in that case only a tautological expression of the sensation itself." And Mr. Mill, the most recent and certainly one of the ablest of metaphysical writers, thus expresses the same view. In justifying the assertion that sensations are as truly states of mind as thoughts, emotions, and volitions, he says: "It is usual, indeed, to speak of sensations as states of body, not of mind. But this is the common confusion of giving one and the same name to a phenomenon and to the proximate cause or conditions of the phenomenon. The immediate antecedent of a sensation is a state of body, but the sensation itself is a state of mind. If the word mind means anything it means that which feels. If we allow ourselves to use language implying that the body feels, there is no reason against being consistent in that language and saying that the body also thinks."* The change which we call sensation is, in fact, neither more nor less than the consciousness of an impression. If we wish to describe a change taking place in the central organs of the nervous system, of which the individual does not necessarily experience consciousness, we cannot therefore employ the term *sensation* without contradicting its received acceptation. Yet Dr. Arnold accuses Dr. M. Hall of inconsistency in making the term sensation imply consciousness; and his chief argument seems to be, that Plato maintained that sensations are dependent upon the organs of sense, having special relations with the peculiar structure of these organs, so that neither of them can minister to the sensation receivable through the other ; an argument of which we fail to perceive the applicability. Dr. Arnold, however, thinks that it enables him to refute many of the doctrines of the reflex theory. Yet, as will presently appear, the idea which he would substitute is essentially the same, the difference being chiefly in the language in which it is expressed.

In regard to the term *perception*, there is more latitude among scientific writers. Some employ it to designate a process distinct from sensation and consequent upon it; this process being the formation of that general notion respecting the nature of the object causing the sensation, which it is certain the sensation itself does not convey, and which is, in man at least, a complex process, involving acts of memory and association and comparison, although often, through repeated experience, performed in an incredibly short period of time. But some writers have employed it in a much looser sense, almost identifying it with sensation; whilst others have employed it in a still lower meaning, implying by it the simple reception of an impression, not even involving consciousness. This was the sense in which it was used by Glisson, who describes irritability (or what we now term vitality) as a *perceptio*, and speaks of sensation as a *perceptio perceptionis*, a definition which, interpreted as the author meant it to be, comes very near we believe to our own, the consciousness of an impression. It is in the same sense that the term is

* System of Logic, vol. ii. p. 497.

employed by the late ingenious Dr. Fletcher, who even extends it to the actions of different kinds of inorganic matter upon each other. "The thing acted on," he remarks, "must, even in this last case (that of inorganic matter), have *perceived* the influence of the thing acting, or it would not have obeyed this influence; the organized tissues in general must, in the second case (that of the ordinary phenomena of organic life,) have *perceived* the influence of the stimuli which they obey, or they would never have manifested this obedience; and it is only in another kind of *perception,* enjoyed by some only of the organized tissues, that sensation appears to consist, a perception so far elevated above that in which mere irritation (which always precedes it) consists, that a certain degree of consciousness attends it. *The being manifesting it not only perceives, but perceives that he is perceiving.*" This passage serves as a commentary on many expressions of Dr. Arnold and other German writers, which would otherwise be obscure. It shows that we are often to understand by the term *perception,* as used by them, merely the reception of an impression, not in the least involving consciousness. We shall hereafter see that Dr. Arnold attributes to the spinal cord the power of *perceiving* the impressions of external stimuli, and of *reacting* in accordance with them. We should like to know in what respect the property which he thus attributes to the spinal cord differs from the *excitabilité* of Flourens or the *reflex function* of Dr. M. Hall.

With these preliminary cautions we shall now follow Dr. Arnold through his examination, which consists of ten chapters. In the first he sets forth the *reflex theory of Dr. M. Hall and Prof. Müller.* The two theories, however, have many points of dissimilarity; and we shall confine ourselves for the present to that of Dr. M. Hall, reserving for a future occasion some critical remarks on that of Müller. Dr. M. Hall may fairly complain that his theory is not fairly stated by his antagonist; for Arnold has evidently drawn his chief materials from Dr. Hall's *first* memoir, and adopts positions which Dr. Hall has abandoned, besides omitting much important evidence subsequently adduced. Hence he commits the error of stating that Dr. Hall recognizes the four following species of muscular motion: 1, voluntary motion, having its origin in the brain; 2, respiratory motion, having its origin in the medulla oblongata; 3, involuntary motion, resulting from the application of stimuli to the muscles themselves; and, 4, reflex action, performed through the spinal cord and its nerves. This is the classification adopted by Dr. Hall in his first memoir; the authority of Sir C. Bell (it would seem) having led him to consider the respiratory movements under a distinct category. But before the publication of his second memoir, he had satisfied himself that they belong to the same class with the other reflex movements, with which he therefore associates them. Among the chief omissions in the representation of Dr. Hall's theory is that of due reference to the cases in which accident or disease has furnished the means of unequivocally proving, by reference to the human subject, that sensation is *not* a necessary link in the chain by which reflex actions are performed. Here again we find an absence of that reference to Dr. Hall's subsequent writings which his critic ought certainly to have made, before passing judgment upon opinions which were evidently crude, and intended by their author to be tested by subsequent research. We have ourselves always regarded these cases as furnishing more satisfactory proof of the truth of that part of Dr. Hall's reflex theory which respects the absence of sensation, than any experiments on animals can do; for the inferences from these *must* be open, until they are thus confirmed, to the objection that we cannot be sure that consciousness and sensibility do not exist in the spinal cord, so long as the reflex movements can be excited through it.

The second chapter professes to give the *Views of several physiologists of modern times on the reflex theory.* Of these Volkmann is the first quoted; but as a summary of his opinions, with some critical remarks upon them, has been given in a former Volume (vol. VI. pp. 211-18), we need not here go over the ground again. The views of Nasse are of a similar character: Carus objects to the term reflex function, as implying a peculiar property of the spinal cord; and also to the assumption (as he regards it) of a system of excito-motory nerves distinct from the sensori-volitional; but he admits that the spinal cord contains, not merely primitive nervous fibres, but also a pulpy substance, which, like the gray matter of the brain or of the ganglia of the sympathetic, has the power of receiving impressions and of reacting in accordance with them. To this he attributes the movements which are performed by parts whose communication with the brain is interrupted. We confess ourselves at a loss to see in what essential respect this doctrine can be said to differ from that of Dr. Hall. Other criticisms, very much to the same effect, are extracted from the writings of Valentin, Kürschner the translator of Dr. Hall's memoirs, Prof. F. Arnold, and Klencke. Mr. Grainger's observations are very slightly alluded to; but more attention is given to the criticisms of Dr. Griffin, (Medical Gazette, vol. xxiv.) who particularly objects to that portion of Dr. Hall's views which respects the absence of sensation as a necessary link in the production of reflex movements. We have ourselves carefully weighed Dr. Griffin's arguments, but we fail to perceive their cogency. There appears to us to be much looseness in the manner in which the term sensation is employed; and in several portions of the paper we fancy that Dr. Griffin's ideas do not differ greatly from our own; but that he expresses them in different, and what seems to us incorrect, language.

In the third chapter our author takes the trouble to adduce several instances of the *Early use of the term reflex*, for the sake of proving that there is no novelty in Dr. Hall's application of it. We are not aware that he ever claimed any. No one knows better than Dr. Hall that a reflex function had been attributed to the central organs of the nervous system by Haller, Unzer, Prochaska, and many later physiologists; but he claims as his own the limitation of this function to the *true* spinal cord.

The title of the fourth chapter is the *Early knowledge of the facts on which the reflex-theory is founded.* We find in it nothing of any importance which our former historical summary did not include, and shall therefore pass it over without further notice. Nearly the same may be said of the fifth chapter, entitled *Reflex theories of physiologists before Marshall Hall.* An important fact, however, is mentioned respecting Haller, which we had overlooked, namely, that he expressly distinguishes the movements resulting from muscular irritability from those which take place when the head has been cut off or the brain removed; these last continuing to manifest themselves as long as the spinal cord and medulla oblongata remain uninjured. (Elementa Physiologiæ, vol. iv. p. 337.) It is evident, then, that Haller was well aware of the dependence of the reflex movements upon the spinal cord alone; but that he could have had no clear idea as to the nature of the reflex action of this organ is evident, from the fact of his unwillingness to admit a distinction between the afferent and efferent (in common language, the sensory and motor) nervous fibres. It would appear, however, that Unzer came nearer the truth; and that in his Physiology, published in 1771, he expressed nearly the same opinions as those put forth not long afterwards by Prochaska, who is not quoted in Dr. Arnold's historical summary. The ideas of Whytt, Sir G. Blane, Legallois, and Burdach are successively adduced in support of the author's position that there is nothing new in the doctrine of the reflex function; but Flourens, who in our opinion approached it most nearly, is omitted. Referring those of our readers who may feel interested in the subject to the retrospective view of it which we formerly gave, we may here repeat that it cannot be questioned that the reflex theory, limited to the expression of it given by Dr. Hall in his first memoir, contained little that could be regarded as a decided advance upon the doctrines of previous physiologists, although none of them had ever exhibited them in the same combined form, or developed their extensive application. But it is on his conception of a system of nervous fibres appertaining exclusively to the *true spinal cord*, and ministering to the *reflex actions alone* (unless we admit them to be the instruments of the emotions also), physiologically distinct therefore from those which are concerned in sensation and voluntary motion, that Dr. Hall has rested his claim as a discoverer; and this claim we think it impossible to impugn. The only question is with respect to the validity of the conception; and on this we have already expressed our opinion.

The sixth chapter is on the *Distinction between the various kinds of muscular motion, according to Marshall Hall and John Müller.* The author's criticism upon Dr. Hall appears to us to be here chiefly of a verbal character. He objects, and with reason, to Dr. Hall's use of the term *involuntary* as designating the class of movements which depend upon the immediate application of the stimuli to the muscular fibre; since a large number of movements, including all those of the reflex system, depend upon other conditions. Dr. Hall obviously intended to refer to the movements of those muscles which have never anything else than an *involuntary action*,—the heart, for instance, and the muscular coat of the alimentary canal. The other muscles of the body are voluntary or involuntary in their action, according as the stimulus is conveyed to them by the sensori-volitional, or the excito-motor system of nerves. With the criticisms upon Müller's classification of muscular movements, we have at present nothing to do.

In the seventh chapter are considered the *Signification of the term reflexion*, and the nature of the *operation of the spinal cord concerned in reflex motion.* This part, also, of Dr. Arnold's criticism is rather verbal than philosophical. Dr. M. Hall has never attempted to specify the precise action which the spinal cord performs, when it gives rise to a motion in obedience to the stimulus of an external impression; he has merely employed the term reflex-function, as a general designation for that peculiar power in the organ, to which are due a large number of phenomena agreeing in their essential conditions; and we cannot see what objection can be taken to this. In his later writings we think that he has laid himself open to criticism of this kind, in his employment of the term *vis nervosa*, as if it designated a known or appreciable entity. Dr. Arnold, presuming that the advocates of the reflex-theory imply by the term *reflexion* somewhat the same action on the nervous principle as do physical philosophers in regard to light, thus defines it: "We understand by it a *throwing back* of the nervous principle in the spinal cord, without at the same time admitting the existence of a change similar to sensation in the brain, or an effort in the spinal cord analogous to the will." We do not see why it was necessary to go in search of such a definition, when Dr. Hall has on so many occasions expressed his idea of the reflex function with the utmost perspicuity. Dr. Arnold then finds fault with the advocates of the reflex-theory, for not explaining what takes place in the spinal cord when motions are excited through its agency; but we do not see what right he has to do so, until he has explained what takes place in the brain when a sensation is produced there, or a voluntary motion originated. Certainly it behoves the advocates of the new system to be very guarded in the language they employ, so that they may not appear to assume that which they do not claim to know; whilst, on the other hand, they do not leave unexplained any phenomena for which their principles will account. We do not see how any charges of this kind can be fairly brought against Dr. M. Hall; but certainly Dr. Arnold makes out a strong case of inconsistency against Professor Müller. Into this, however, we need not now enter.

The title of the eighth chapter is *Sensation and its seat*, and also its *connexion with reflex motions.* Dr. Arnold commences with the argument for the possibility of sensation without consciousness, on which we have already made some observations. He then goes on to show that Dr. M. Hall's experiments, on which that physiologist originally rested his assertion that sensation is not necessarily involved in reflex actions, are not conclusive; and on this head, as we before observed, we agree with him. But Dr. Arnold does not refer to those other and more convincing proofs which Dr. Hall's second memoir contained. And he considers that conclusion drawn from Dr. Hall's experiments respecting the absence of sensibility in parts of the body cut off from connexion with the brain by section of the spinal cord is true only when the term sensation is restricted to consciousness united with perception (Wahrnehmung.) We believe that we are here to understand this last word in the higher sense in which the term perception is employed in this country; and to regard our author as meaning that the spinal cord is sufficient for what *we* term sensation, and that communication with the brain is only required for the mental changes consequent upon it. If this be his view, it is entirely contradicted by the cases of injury of the spinal cord in the human subject, to which we have already so frequently alluded. But if Dr. Arnold uses the term in its lower sense, he then says nothing different from Dr. M. Hall, namely, that when communication with the brain is cut off the consciousness of impressions made upon the parts of the body thus separated is abolished.

The ninth chapter contains a critical view of the opinions of Dr. M.

Hall, and Professor Müller on the *physiology of the spinal cord, so far as it stands in relation to the reflex theory.* Here, as elsewhere, we shall restrict ourselves to the comments upon the views of Dr. Hall. As already stated, the organ commonly designated as the spinal cord is believed by Dr. Hall to be composed of two parts: the intravertebral cord, which connects the nerves with the cerebrum; and the true spinal cord, the axis or centre of the excito-motor system of nerves. He confesses his inability, however, to isolate these parts anatomically in vertebrated animals; but he thinks it probable that they may be found distinct in the invertebrata; and he adds, "but if the existence of a *distinct* anatomy of the excito-motory system be doubtful, that of the *blended* anatomy, and that of the distinct physiology, pathology, and therapeutics of that system, are perfectly obvious." Dr. Hall has never claimed for himself the discovery of the *structural distinctness* of the two systems; but has constantly put forth his arguments in proof of their *physiological* distinctness. Now we cannot but regard this distinction as more verbal than real. The question after all comes to this. Do the same nervous fibres minister to the reflex function and to sensori-volitional changes; or are these performed by different sets of fibres? If they *are* performed by the *same*, then the two systems cannot be regarded as physiologically distinct; and many of Dr. Hall's arguments in favour of the reflex-function fall to the ground. If the two sets of actions are performed by *different* fibres, then the systems *are* structurally distinct; though the two classes of fibres may be everywhere bound up in the same sheath, and may not be distinguishable at their central origins or terminations. It is not getting out of the difficulty, in which the advocates of the reflex-function are placed, by the want of a clear demonstration of the excito-motor system in the spinal cord of vertebrata, to say that such a demonstration is not required, since only a *physiological* distinctness is claimed; and we do not think that Dr. Hall has done wisely in leaving the question in this state. He certainly lays himself open, thereby, to the criticism of Dr. Arnold; who asserts that all the facts adduced by Dr. Hall may be explained upon the property of the spinal cord long known to physiologists; and upon the supposition, that the conveyance of sensory impressions and of those destined to excite reflex actions, on the one hand, and the transmission of voluntary and reflex motor impulses on the other, are accomplished by the same nerves. The following are his principal arguments, on which we shall comment as we proceed.

1. The skin, which is the organ for the reception of sensory impressions of such variety and acuteness, is also the organ through which the stimuli that excite reflex actions make their strongest impression. Hence it may be concluded that the nerves which go to the skin are both sensory and excitor.

2. If we admit the existence of distinct sensory and excitor nerves, we must admit that both kinds are distributed even in the minutest ramifications; since every portion of the skin is capable of receiving and transmitting both sensory and excito-motor impressions. And in the same manner we must admit that the minutest twigs supplying the muscles contain a cerebral fibre and a spinal fibre.

We own ourselves unable to see the force of the objections adduced in these arguments. There are very obvious reasons why the general exterior surface of the body should be endowed alike with the capability of receiving sensory impressions and the stimuli to excito-motor impulses; and there can hence be drawn, therefore, not the shadow of an argument against the distinctness of the two sets of nerves. If we *universally* found sensibility and the power of exciting reflex actions associated in the same degree, there might be then more ground for the assumption that they act through the same nerves; but this is by no means the case. For whilst irritation of the *trunk* of a sensory nerve occasions acute pain, it does not excite reflexions in anything like the same degree; and sensibility is often abolished by disease, when the reflex actions are unusually excitable. Knowing that the minutest ramifications of the cutaneous and muscular nerves almost invariably (perhaps invariably,) contain two or more ultimate fibrillæ, we cannot see any force in the second of Dr. Arnold's arguments; and shall therefore at once pass on to the next.

3. When the posterior roots of the nerves going to the posterior extremities have been divided, not only is sensibility abolished in these limbs, but the excitability to reflex actions; and when nux vomica has been given to the animal, no tetanic spasms occur in these parts. In support of this last assertion some interesting experiments are detailed.

As it is universally believed, by the advocates of the reflex doctrine, that the excitor fibres are bound up with the sensory in the posterior roots of the spinal nerves, we cannot see the least force in the first part of this argument; since, as Dr. M. Hall has frequently shown, the reflex actions cannot be excited if the continuity of the nervous circle, consisting of the excitor nerve, the spinal cord, and the motor-nerve, be anywhere interrupted. The absence of the tetanic spasms which succeed the administration of nux vomica from limbs whose sensory nerves have been divided, is an interesting fact, but one not in the least militating against the doctrine. It shows that the effect of the poison is to produce a high degree of *excitability* of the spinal cord; but that the convulsive action is immediately dependent upon some stimulus, (perhaps of the slightest possible nature,) communicated through the excitor nerves. Hence in this artificial tetanus, the spasms are not of central but of peripheral origin.

4. The next argument, or rather series of arguments, is drawn from other effects of nux vomica. Dr. Arnold considers that the fact of its exalting at the same time the sensibility and the excitability is a proof that the excitor and sensory fibres are the same; and that the following points should be kept in view in reasoning upon it. A. The increased susceptibility to irritation, from the action of nux vomica, still exists after the removal of the head or brain, so that convulsive movements or rigid spasm occur, especially in amphibia; and this happens even if the nux vomica be not applied until some time after the removal of the brain. It hence appears that the medulla oblongata and spinal cord are capable of being specifically acted on by this irritant, quite independently of the brain. B. After the injury or partial removal of the medulla oblongata, however, the (so called) reflex motions are much weaker, and sooner come to an end, than after mere decapitation of the animals. The same thing occurs with respect to the action of nux vomica. It appears from Dr. Arnold's experiments on frogs, that the removal of one lateral half of the medulla oblongata does not suspend the excitability of the spinal cord by nux vomica, but diminishes its duration; the removal of a small portion of the upper extremity of the medulla oblongata seems to delay the occurrence of the tetanic spasms occasioned by the nux vomica, and renders the convulsive movements less marked (especially in the posterior extremities) as well as more transient; whilst after the division of the medulla oblongata transversely, the tetanic spasms either do not present themselves at all, or but very slightly. c. After the complete removal of the medulla oblongata, the reflex motions diminish more evidently, both as to energy and duration, than after mere injury of that part. This is also the case with the tetanus occasioned by the application of nux vomica previous to the removal of the medulla oblongata. d. The medulla oblongata, which is the channel through which the sensations of a large part of the body are produced, is the point on which the effects of nux vomica are chiefly manifested. If the nux vomica be not applied until after the complete removal of the medulla oblongata, no convulsive movements, according to Dr. Arnold, are produced by it; whilst if the removal be delayed until after the nux vomica has been applied, and the tetanic state resulting from it has set in, the tetanus then continues.

From these facts it might be concluded, that the spinal cord has no independent powers of its own; and that it reacts only so far as it has received a *charge* from the medulla oblongata. Such a view, however, as Dr. Arnold admits, could be only partially correct; since reflex motions can be excited in portions of the spinal cord that have been long completely separated from the medulla oblongata; and there are various agents which are capable of increasing its excitability, so as to give rise to violent spasmodic actions, after the complete removal of the medulla oblongata. It is curious that strychnine, when employed in its isolated state, should be one of these. We must avow ourselves at a loss to comprehend what bearing these experiments have upon the question of the distinctness of the excito-motor system of nerves; they only point to what is certainly a curious fact,—that the influence of nux vomica is primarily exerted upon the medulla oblongata, and that it extends from this to the rest of the spinal cord.

In the last chapter, entitled "Facts which the observation of nature presents in regard to the so called reflex-function, and inferences from these," we have a summary of Arnold's own researches and conclusions, of which we shall give a full translation, as well in justice to the author, as with the desire to show how the subject is treated among learned physiologists of the German school:

"Unprejudiced observation of the so-called reflex-motions in decapitated animals, presents the following results.

"1. If the convulsive movements, which often follow the decapitation, are over, or if the animal has recovered itself from the paralytic state which frequently succeeds the operation, the subsequent movements are such as result from the application of external stimuli. It is true that, in decapitated frogs, spontaneous (selbständige) movements sometimes occur; but they only take place when the frogs find themselves in unwonted positions, and they result from their endeavours to attain their ordinary position, or one more convenient. Such movements, however, are only spontaneous in appearance; for they are nothing else than the reaction of the bodies played upon.

"2. The motions of decapitated animals, which result from the action of external stimuli, bear the character of *adaptiveness* in themselves, and, when in combination, they indicate internal combination and harmonization. We may generally see in these movements, that their object is to remove the source of the external irritation; and frequently for the attainment of this object a powerful movement is executed. If, for example, a decapitated frog be laid hold of in the back with the forceps, one of the hind-legs is violently moved to the place, and grasps the forceps. Frequently an unmistakeable purpose shows itself in the movement, that of withdrawing itself completely from the stimulus. The animal extends the irritated limb, and the frog jumps away, when the stimulus operates with sufficient power.

"3. The so-called reflex motions are more energetic in decapitated than in undecapitated amphibia. It frequently happens that, in undecapitated frogs, no external stimuli produce motions, when for instance the animals perceive that they cannot immediately withdraw themselves from the stimulus; but after cutting off the head, or removing the brain, determinate and adaptive motions generally follow the action of an irritant on the skin, as soon as the animal has recovered its tranquillity, and its irritability has become re-established.

"4. The liveliness of the movements is increased by those agents which suspend or repress the activity of the brain. If opium be given to an undecapitated frog, it is the result of my experiments that, together with the state of stupor, which prevents the exercise of the will and the free selection of its movements, there is an increased susceptibility in the skin to the action of external stimuli, and consequently a greater liveliness in the so-called reflex motions; there even follow tetanic symptoms, like those resulting from the operation of nux vomica. But in decapitated amphibia, or in those from which the brain has been removed, opium in similar doses no longer increases the irritability of the skin or the movements excited by external stimuli.

"5. Such influences as possess a specific effect in stimulating the medulla oblongata and spinal cord, nux vomica for instance, exalt the sensibility in uninjured animals, and in the same way heighten in decapitated animals the susceptibility of irritation, especially in the skin, and hence aggravate the movements which follow external stimuli, the so-called reflex actions.

"6. The character of the movements which present themselves in decapitated animals depends chiefly upon the state of the spinal cord induced before their decapitation, by influences derived from the brain or the medulla oblongata. They are also modified, in some degree, as to direction, strength, activity, and duration, by the strength or peculiarity of the irritating cause. Thus it is well known that many animals, especially birds, will continue to run, after decapitation, if their heads be cut off whilst they are running. [The facts respecting the different action of nux vomica, according as it is introduced before or after the removal of the medulla oblongata, are here again introduced; and it is admitted that the circumstance of tetanus being induced by strychnine applied after this operation, is an argument for the independence of the spinal cord.]

"7. The so-called reflex motions do not cease to affect the body generally, though the spinal cord be cut through on one side even to the median line, or though a piece of it be entirely removed from one side. In like manner, a longitudinal division of the spinal cord does not prevent the extension of these motions to all the muscles on both halves of the body, provided that some part of the spinal cord remains undivided on the median line.

"8. Section of the posterior roots of the spinal nerves checks the so-called reflex motions. If the nerves of the posterior extremities be thus partly separated, and the spinal cord itself be divided transversely in the middle of the back, the posterior extremities will remain perfectly motionless, which is not the case if the sensory nerves be left without injury.

"9. The strength and extension of the motions from irritation, or the so-called reflex movements, depends on the degree of excitability and on the strength of the irritating cause. But when the susceptibility to irritants is considerably increased, the motions lose the character of adaptiveness, and present that of spasm.

"10. The so-called reflex actions are chiefly produced by irritations of the skin, conjunctiva, and the mucous membrane of the nose, mouth, and throat. No determinate adaptive movements are produced by irritation of the thoracic or abdominal viscera. Even after irritation of the muscles and nervous trunks, only spasmodic twitchings take place, without any character of adaptiveness or harmonious accordance."

From these facts, Dr. Arnold thinks that he is warranted in drawing the following conclusions:

"1. A power of being influenced* by external irritants has its seat in the spinal cord, in a certain degree independently of the brain and medulla oblongata, *the perception-power of the spinal cord.*

"2. This power in the spinal cord relates not merely to the irritant in general, but also to the nature, the degree, and the locality of it; but the faculty of perception allied with consciousness is wanting.

"3. With the perception-power stands in next relation that faculty of the spinal cord, by which it reacts in accordance with the excitement produced by impressions, so as to perform adaptive, combined and harmonious movements; *the reaction-power of the spinal cord.*

"4. These motions are evidently adaptive and harmonious; but they want the character of freedom. They are not external manifestations of a will.

"5. The spinal cord possesses in a slight degree only the faculty of giving origin to spontaneous motions. If in decapitated animals self-originating motions do occur, they are principally the result of a disposition or excitation, which the spinal cord has received from the brain or medulla oblongata previously to the decapitation.

"6. The degree of the perception-power of the spinal cord depends on a peculiar disposition (Stimmung) of this organ, which is induced in it principally by the medulla oblongata, and which, without it, can be manifested by this organ only in a very slight degree. The same may be said of the rapidity and energy of the motions excited by external irritations.

"7. The disposition thus produced in the spinal cord remains in it for some time, even when it is separated from the brain and medulla oblongata; and even in separated parts of it.

"8. The change which takes place in the spinal cord during the reception of external impressions, and the determination of movements consequent upon these, is analogous to that which takes place in the brain during conscious sensations and voluntary motions; only that clear consciousness (klares Bewusstsein) and freedom of will are wanting to it, whilst the character of adaptiveness and of harmonious accordance appertains to it in the highest degree.

"9. The impressions (Eindrücke) which the central organs receive through their nerves produce in them a disposition corresponding to the quality of the impression; depending both on its own nature and on the nerve by which it is received and conducted to the central organs of the nervous system; whereupon these organs react in a corresponding manner.

"10. A mere transference of the nervous principle from sensory to motor fibres does not take place in the spinal cord. The term *reflex-function* does not indicate what really takes place in the organ during the motions occasioned by external irritations.

"11. With regard to the conducting faculty (Leitungsvermögen) of the spinal cord, observation goes to show that it is in its *totality* that it imparts the disposition it has received, either from the brain and the medulla oblongata on the one hand, or from the nerves on the other. After the arguments formerly

* This we believe to be the real meaning of the original: "Ein Vermögen, aüssere Reize inne zu werden, hat in dem Rückenmarke," &c. The author does not seem to imply what *we* term *sensation;* as appears from the next position.

adduced, it can no longer be admitted that the fibres of the spinal cord conduct impressions in an isolated manner, as do those of the nerves.

"12. It is not the number of the muscles moved that is determined by the central organ; but the object which is to be attained. A *Clavier-theory*,* such as has been introduced of late years, has no facts in favour of it, and many against it."

It will be evident, we think, to any candid reader of the preceding extract, that Dr. Arnold admits the essential principles of the reflex-theory whilst apparently dissenting from it. Many of the objections he adduces bear rather against the ideas he has himself attached to it, than against the theory itself, as propounded by Dr. Hall, and sustained by his followers. Thus we are not aware that any one has spoken of "a mere transference of the nervous principle from sensory to motor fibres" as the entire change to which the spinal cord is subservient in reflex action; or has attempted to explain the operations which really take place there. It must be admitted that some of Dr. Hall's expressions regarding the course of the *vis nervosa* along the incident nerves, and its reflexion by the spinal cord along the motor nerves, do give an appearance of validity to Dr. Arnold's objections; and we have always regretted that Dr. Hall should use language which appeared so capable of misrepresentation, whilst it did not give the least assistance in the explanation of the phenomena to which his theory applies. But Dr. Hall knows, as well as we do, that the true spinal cord has properties peculiar to itself, and that it is something very different from a mere centre of communication between incident and motor fibres. Although it might seem quite possible to explain the *ordinary* reflex movements by such communications, especially since the incident and motor nerves, through which these are executed, are almost invariably connected with the same portion of the ganglionic centre, yet this mode of explanation can scarcely be regarded as applicable to those abnormal convulsive actions in which almost every muscle in the body is thrown into violent action from the effect of a stimulus conveyed through a single excitor nerve. This is obviously the case, for example, in traumatic tetanus, and in hydrophobia, where we can distinctly trace the source of the irritation. In epilepsy and hysteria, the local cause of the convulsive actions is often more obscure, but it is not the less real. But in these disorders the convulsive movements are not limited to particular groups of muscles, such as might be imagined to be connected with the incident nerves that excite them; they affect the whole body, and especially the respiratory system. It is evident that in these cases, as in the artificial tetanus induced by strychnine, the whole spinal cord is in a state of undue irritability, so that any stimulus conveyed to it will react, not through one or two nerves only with which the incident fibres may have the closest relations, but through the gray matter of the cord, which gives to it its peculiar character as a ganglionic centre, and constitutes the medium of communication between the incident and motor *systems* of fibres. We know nothing of the operations of this gray matter, any more than we are acquainted with the precise mode in

* Dr. Arnold here refers to the doctrine propounded by Müller, that the will acts upon the motor nerves through the brain and medulla oblongata, as a performer on a keyed instrument produces what sounds he pleases by striking the appropriate keys.

which changes are propagated along the white fibres; but we have good reason to believe that whilst each *fibre* of the *white* matter is completely isolated from the rest so as not to communicate to it any influence which it is conveying; the ultimate constituents of the *gray* matter are not so isolated but that they are able to propagate to each other (to a greater or less extent, according to circumstances) the changes they are undergoing. Now we will suppose that a certain tract of gray matter intervenes between the termination of a given incident nerve and the origin of the motor nerve by which it ordinarily reacts; the stimulus conveyed by this incident nerve usually produces such a change in the tract of gray matter, that it excites the respondent motion, and that only. But supposing the spinal cord (by which we understand its gray matter or ganglionic substances) to be in a state of unusual irritability, then the operation of this stimulus will spread itself to other parts of the gray matter, perhaps even to a considerable distance, and will produce respondent motions in a great variety of muscles. This hypothesis appears to us to account sufficiently well for observed phenomena to possess a claim to be received as their explanation, until something more definite shall be known respecting the real changes which take place in the nervous system, during its active operations. It will be found, we think, perfectly conformable with all the facts adduced by Dr. Arnold, against what he erroneously believed to be the essential principles of the reflex-theory.

---

